# Supplementary material for: Integrated Placental Modelling of Histology with Gene Expression to Identify Functional Impact on Fetal Growth
Source: Cells. 2023 Apr 6;12(7):1093. doi: 10.3390/cells12071093 (PMC10093760; doi:10.3390/cells12071093)
Supplement: Supplementary file 1 [file cells-12-01093-s001.zip › cells-2286276-supplementary.pdf]

SUPPLEMENTARY INFORMATION

Supplementary Table S1: Full phenotype clinical data.

ID	Status	Medical history	Pregnancy complications	Maternal age quartile (years)	Ethnicity	Maternal booking BMI	Delivery mode	Gesta tion	Birthweight (g)	Infant sex	Placenta weight (g)	Placenta/ BW ratio (%)	Analysis comments
Control 1	Control			31-35	Asian	34	Vaginal delivery	39+6	3200	Male	442	13.8	
Control 2	Control			36-40	White	21	cat4 CS	39+1	3690	Female	516	14.0	
Control 3	Control			21-25	White	20	Vaginal delivery	39+0	3800	Female	534	14.1	
Control 4	Control			31-35	White	23	cat3 CS	41+1	3970	Male	509	12.8	
Control 5	Control			41-45	White	27	cat4 CS	39+1	3960	Female	481	12.1	
Control 6	Control			36-40	White	24	cat4 CS	39+1	2893	Female	413	14.3	
Control 7	Control			36-40	White	23	Vaginal delivery	39+0	3618	Male	491	13.6	
Control 8	Control			36-40	White	23	cat4 CS	39+1	3140	Male	398	12.7	
Control 9	Control			31-35	White	28	Vaginal delivery	39+5	3510	Male	526	15.0	
FGR 1	Late FGR			31-35	White	23	cat3 CS	37+0	1410	Male	160	11.3	

FGR 2	Late FGR	Hypothyroid		31-35	White	22	Vaginal delivery	37+5	2510	Female	346	13.8	
FGR 3	Late FGR	Migraines		31-35	Chinese	22	cat2 CS	37+2	2560	Male	380	14.8	
FGR 4	Late FGR		Low platelets	26-30	Afro Caribbean	22	cat2 CS	38+2	1985	Male	380	19.1	
FGR 5	Late FGR			31-35	Chinese	20	Vaginal delivery	39+4	2720	Female	360	13.2	
FGR 6	Late FGR		Gestational diabetes	31-35	Asian	38	cat1 CS	38+4	2396	Male	355	14.8	
FGR 7	Late FGR			36-40	Afro Caribbean	28	cat3 CS	39+0	2750	Male	404	14.7	Not run for qPCR

CS = caesarean section. Cat1 = Immediate threat to the life of the woman or fetus, Cat2 = Maternal or fetal compromise which is not immediately life-threatening, Cat3 = No maternal or fetal compromise but needs early birth, Cat4 = Birth timed to suit woman or healthcare provider. (NICE guideline 192: Caesarean birth, published 31 March 2021).

Supplementary Table S2: Morphological differences in the placenta by sampling location.

	Control (N=9)			FGR (N=7)		
	**Central**	**Peripheral**	**P-value**	**Central**	**Peripheral**	**P-value**
**Volume density (cm^3/g)**						
Intervillous space	0.39±0.02	0.36±0.01	0.130	0.36±0.02	0.35±0.02	0.577
Stem villi	0.06±0.01	0.06±0.01	0.902	0.06±0.02	0.05±0.01	0.364
Intermediate villi	0.1±0.01	0.13±0.01	0.105	0.08±0.01	0.08±0.01	0.720
Terminal villi	0.42±0.02	0.42±0.02	0.941	0.41±0.03	0.38±0.03	0.526
Syncytial knots	0.02±0.00	0.02±0.00	0.633	0.01±0.00	0.01±0.00	0.591
Fibrosis	0.01±0.00	0.02±0.01	0.443	0.07±0.02	0.13±0.03	0.091
Trophoblast	0.33±0.02	0.31±0.01	0.492	0.34±0.02	0.33±0.04	0.818
Stromal	0.53±0.03	0.57±0.02	0.239	0.45±0.04	0.46±0.06	0.865
Capillary	0.14±0.01	0.12±0.01	0.139	0.21±0.03	0.21±0.03	0.963
**Volume (cm^3)**						
Intervillous space	187.75±10.19	171.7±9.96	0.277	121.96±11.52	116.92±10.14	0.748
Stem villi	27.82±5.18	27.85±2.89	0.100	23.24±6.3	17.1±2.58	0.385
Intermediate villi	49.91±4.81	60.89±5.94	0.170	24.77±2.83	26.19±2.23	0.700
Terminal villi	199.23±8.7	202.24±13.7	0.855	141.08±17.48	129.51±15.51	0.630
Syncytial knots	8.89±0.9	7.89±1.12	0.495	2.24±0.66	2.63±0.82	0.720
Fibrosis	3.9±1.93	7.87±4.69	0.446	26.28±6.89	47.77±9.57	0.094
Trophoblast	156.49±6.76	150.51±8.25	0.583	117.28±13.22	112.13±15.07	0.802

Stromal	254.68±18.75	271.49±12.97	0.472	150.39±19.02	156.07±25.00	0.860
Capillary	67.72±5.88	56.89±5.04	0.181	73.05±14.18	72.51±13.58	0.979
**Barrier thickness (μm)**						
Arithmetic mean	2.84±0.08	2.82±0.10	0.885	2.84±0.19	2.86±0.18	0.940
Harmonic mean	2.14±0.07	2.15±0.07	0.918	2.24±0.15	2.24±0.14	0.987
Thickness uniformity index	1.33±0.02	1.31±0.02	0.508	1.47±0.08	1.48±0.08	0.936
**Surface density (cm^2/cm^3)**						
Villi	613.67±27.94	607.22±24.07	0.863	634.49±43.19	626.07±47.42	0.898
Fetal capillary	318.63±27.71	277.88±13.62	0.206	406.06±47.11	397.78±51.52	0.906
**Surface area (m^2)**						
Villi	15.32±0.97	15.99±0.92	0.622	10.87±1.63	10.3±1.51	0.801
Fetal capillary	7.89±0.75	7.32±0.44	0.519	7.11±1.31	6.67±1.2	0.807
**Theoretical diffusion capacity (cm^2/min/kPa)**	94.95±5.96	95.64±4.31	0.926	71.63±11.95	68.89±11.61	0.872
**Specific diffusion capacity (cm^2/min/kPa/g)**	27.15±1.86	27.24±1.23	0.968	30.24±4.58	28.7±3.71	0.798

Supplementary Table S3: Summary tables of errors in all 27 models for each patient.

ONLY PERIPHERAL										
ID	Score	STEREOLOGY & PCR			PCR			STEREOLOGY		
		xgbTree	glmnet	nnet	xgbTree	glmnet	nnet	xgbTree	glmnet	nnet
Control 1	0	0 of 4	0 of 4	0 of 4	1 of 4	1 of 4	1 of 4	0 of 4	0 of 4	0 of 4
Control 2	0	1 of 4	0 of 4	0 of 4	0 of 4	0 of 4	0 of 4	1 of 4	0 of 4	0 of 4
Control 3	0	0 of 4	0 of 4	0 of 4	0 of 4	1 of 4	1 of 4	0 of 4	0 of 4	0 of 4
Control 4	0	0 of 4	0 of 4	0 of 4	4 of 4	3 of 4	3 of 4	0 of 4	0 of 4	0 of 4
Control 5	0	0 of 4	0 of 4	0 of 4	2 of 4	0 of 4	0 of 4	0 of 4	0 of 4	0 of 4
Control 6	0	0 of 4	0 of 4	0 of 4	3 of 4	3 of 4	4 of 4	0 of 4	0 of 4	0 of 4
Control 7	0	0 of 4	0 of 4	0 of 4	0 of 4	0 of 4	0 of 4	0 of 4	0 of 4	0 of 4
Control 8	0	0 of 4	0 of 4	0 of 4	1 of 4	2 of 4	2 of 4	0 of 4	0 of 4	1 of 4
Control 9	0	0 of 4	0 of 4	0 of 4	0 of 4	0 of 4	0 of 4	0 of 4	0 of 4	0 of 4
FGR 1	1	4 of 4	0 of 4	0 of 4	4 of 4	4 of 4	4 of 4	0 of 4	0 of 4	0 of 4
FGR 2	1	0 of 4	0 of 4	0 of 4	0 of 4	0 of 4	0 of 4	0 of 4	0 of 4	0 of 4
FGR 3	1	0 of 4	0 of 4	0 of 4	0 of 4	0 of 4	0 of 4	0 of 4	0 of 4	0 of 4
FGR 4	1	0 of 4	0 of 4	0 of 4	0 of 4	0 of 4	0 of 4	0 of 4	0 of 4	0 of 4
FGR 5	1	0 of 4	0 of 4	0 of 4	0 of 4	0 of 4	0 of 4	0 of 4	0 of 4	0 of 4
FGR 6	1	4 of 4	3 of 4	1 of 4	4 of 4	4 of 4	4 of 4	4 of 4	3 of 4	3 of 4

ONLY CENTRAL										
ID	Score	STEREOLOGY & PCR			PCR			STEREOLOGY		
		xgbTree	glmnet	nnet	xgbTree	glmnet	nnet	xgbTree	glmnet	nnet
Control 1	0	0 of 4	0 of 4	0 of 4	0 of 4	0 of 4	0 of 4	0 of 4	0 of 4	0 of 4
Control 2	0	0 of 4	0 of 4	0 of 4	0 of 4	0 of 4	0 of 4	0 of 4	0 of 4	0 of 4
Control 3	0	0 of 4	0 of 4	0 of 4	2 of 4	2 of 4	0 of 4	0 of 4	0 of 4	0 of 4
Control 4	0	0 of 4	0 of 4	0 of 4	2 of 4	3 of 4	3 of 4	0 of 4	0 of 4	0 of 4
Control 5	0	0 of 4	0 of 4	0 of 4	0 of 4	0 of 4	0 of 4	0 of 4	0 of 4	0 of 4
Control 6	0	3 of 4	2 of 4	1 of 4	3 of 4	3 of 4	3 of 4	0 of 4	0 of 4	0 of 4
Control 7	0	0 of 4	0 of 4	0 of 4	0 of 4	0 of 4	0 of 4	0 of 4	0 of 4	0 of 4
Control 8	0	2 of 4	0 of 4	1 of 4	0 of 4	0 of 4	1 of 4	2 of 4	1 of 4	1 of 4
Control 9	0	0 of 4	0 of 4	0 of 4	1 of 4	2 of 4	1 of 4	0 of 4	0 of 4	0 of 4
FGR 1	1	1 of 4	0 of 4	0 of 4	4 of 4	4 of 4	4 of 4	0 of 4	0 of 4	0 of 4
FGR 2	1	0 of 4	0 of 4	0 of 4	0 of 4	0 of 4	0 of 4	0 of 4	0 of 4	0 of 4
FGR 3	1	0 of 4	0 of 4	0 of 4	0 of 4	0 of 4	0 of 4	0 of 4	0 of 4	0 of 4
FGR 4	1	0 of 4	0 of 4	0 of 4	0 of 4	0 of 4	0 of 4	1 of 4	0 of 4	0 of 4
FGR 5	1	0 of 4	0 of 4	0 of 4	0 of 4	0 of 4	0 of 4	0 of 4	0 of 4	0 of 4
FGR 6	1	4 of 4	4 of 4	4 of 4	4 of 4	4 of 4	4 of 4	3 of 4	4 of 4	4 of 4

CENTRAL & PERIPHERAL										
ID	Score	STEREOLOGY & PCR			PCR			STEREOLOGY		
		xgbTree	glmnet	nnet	xgbTree	glmnet	nnet	xgbTree	glmnet	nnet
Control 1	0	1 of 8	0 of 8	0 of 8	1 of 8	1 of 8	1 of 8	1 of 8	0 of 8	0 of 8
Control 2	0	0 of 8	0 of 8	0 of 8	0 of 8	0 of 8	0 of 8	0 of 8	0 of 8	0 of 8
Control 3	0	0 of 8	0 of 8	0 of 8	1 of 8	2 of 8	2 of 8	0 of 8	0 of 8	0 of 8
Control 4	0	0 of 8	0 of 8	0 of 8	5 of 8	2 of 8	5 of 8	0 of 8	0 of 8	0 of 8
Control 5	0	0 of 8	0 of 8	0 of 8	2 of 8	0 of 8	0 of 8	0 of 8	0 of 8	0 of 8
Control 6	0	3 of 8	1 of 8	0 of 8	6 of 8	7 of 8	6 of 8	0 of 8	0 of 8	0 of 8
Control 7	0	0 of 8	0 of 8	0 of 8	0 of 8	0 of 8	0 of 8	1 of 8	0 of 8	0 of 8
Control 8	0	1 of 8	0 of 8	0 of 8	1 of 8	1 of 8	1 of 8	1 of 8	2 of 8	0 of 8
Control 9	0	1 of 8	0 of 8	0 of 8	1 of 8	0 of 8	0 of 8	1 of 8	0 of 8	0 of 8
FGR 1	1	0 of 8	0 of 8	0 of 8	8 of 8	8 of 8	8 of 8	0 of 8	0 of 8	0 of 8
FGR 2	1	0 of 8	0 of 8	0 of 8	0 of 8	0 of 8	0 of 8	0 of 8	0 of 8	0 of 8
FGR 3	1	0 of 8	0 of 8	0 of 8	1 of 8	0 of 8	0 of 8	1 of 8	0 of 8	0 of 8
FGR 4	1	0 of 8	0 of 8	0 of 8	0 of 8	0 of 8	0 of 8	0 of 8	0 of 8	0 of 8
FGR 5	1	0 of 8	0 of 8	0 of 8	0 of 8	1 of 8	1 of 8	0 of 8	0 of 8	0 of 8
FGR 6	1	8 of 8	7 of 8	5 of 8	8 of 8	8 of 8	8 of 8	6 of 8	7 of 8	7 of 8

Each cell for each patient and 27 models considered (different columns) indicates how many wrong predictions were made for this patient among all 4 or 8 available samples (for example, the entry 0 of 4 means that 0 errors were made among 4 predictions). The cell is highlighted in red if at least one mistake was made for the patient. Third column in the first panel corresponds to the best model, using *nnet* ML algorithm, and both stereology and PCR data for peripheral samples. This model returns only one wrong prediction out of 60 samples considered.

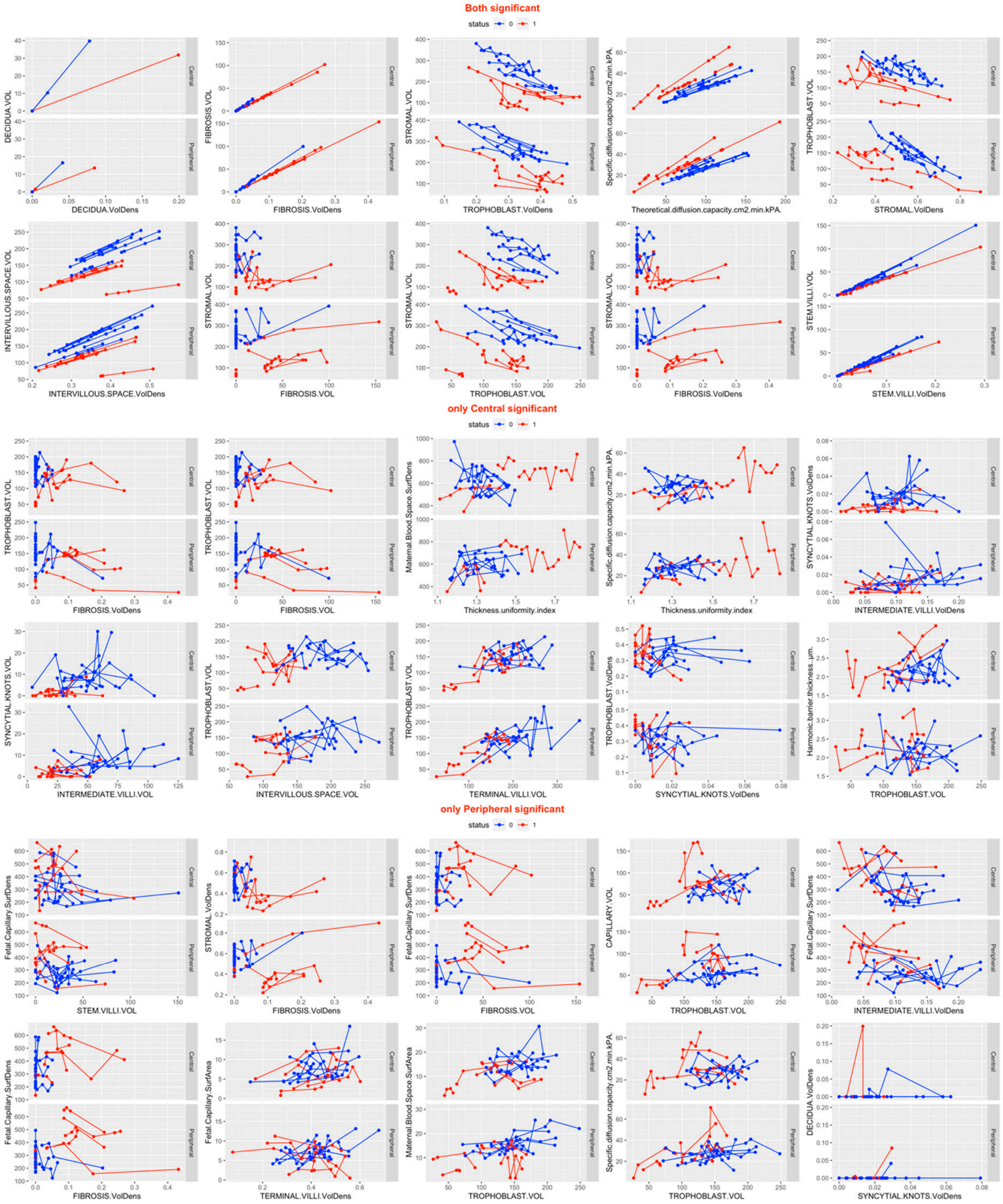

Supplementary Figure S1: Different interdependencies of features for stereological data. Different interdependencies of features for Control (blue colour) and FGR (red colour) on stereological data. Each point is a separate patient measurement, each line connects all measurements for each patient. For many pairs of features, a separation between FGR cases and controls was easily seen, hence, enabling a construction of a successful predictive model.
